# Identifying risk individuals for heart failure diagnosis within two years in the adult population in southern Sweden using gender, age, multimorbidity level, and socioeconomic status

**DOI:** 10.1186/s12872-026-06230-9

**Published:** 2026-07-06

**Authors:** Mia Scholten, Anders Halling

**Affiliations:** Center for Primary Health Care Research, Department of Clinical Sciences, Malmö Lund University, Malmö, Sweden

**Keywords:** heart failure, multimorbidity, probability, age, odds ratio, socioeconomic status

## Abstract

**Background:**

Heart failure (HF) is a common disease among older individuals and is associated with poor quality of life and prognosis. Individuals at risk of developing HF are usually already patients in primary healthcare, but diagnosing HF at an early stage can be challenging. Identifying patients at risk of HF and initiating early treatment is crucial for their outcomes. Using the variables gender, age, multimorbidity (MM) level, and socioeconomic status (SES), we aimed to study the possibility of identifying individuals at high risk of HF diagnosis within two years.

**Methods:**

A longitudinal registry-based study, including 961,190 inhabitants aged from 20 years onwards without a HF diagnosis living in southern Sweden during 2015. Logistic regression was applied to estimate the OR of HF diagnosis within two years by adjusting for the variables gender, age, MM level, and SES. Linear predictions were made based on models by adding these variables in steps. Each model was compared with the previous model using a likelihood-ratio test. The optimal cutoff point for sensitivity and specificity was calculated using the Youden method.

**Results:**

Age had the highest OR of HF diagnosis within two years, followed by MM level, gender, and SES. ROC (Receiver Operating Characteristic) analysis, including these variables in steps, generated an increasing AUC (area under the curve), from 0.5144 to 0.9379. When all four variables were included in the model, an optimal cutoff point according to Youden was established at 1.15%, which predicted the probability with a sensitivity of 87.69% and specificity of 78.48%. The positive predictive value was 4.78%, and the negative predictive value was 99.81% for the whole adult population; for those aged 70 years and older, it was 21.02% and 98.99%; and for those aged 80 years and older, it was 33.62% and 98.09%, respectively.

**Conclusions:**

Age was the most important factor for predicting the probability of HF diagnosis within two years in our study, followed by MM level, gender, and SES. These findings may help identify population groups at increased risk of HF in whom targeted case-finding strategies could be evaluated in future studies.

## Introduction

Heart failure (HF) is a common disease associated with age and constitutes a high healthcare burden globally [1, 2]. Many patients at risk of developing HF are already patients in primary healthcare, but diagnosing HF at an early stage can be challenging. Patients with acute HF are reported to have a poor prognosis, comparable with those experiencing acute myocardial infarction [3]. Patients with stable chronic HF without treatment are also at high risk for fatal complications such as sudden death. Any delay of HF treatment initiation is associated with a worse outcome and is therefore regarded as an important modifiable risk factor, because it is beneficial regardless of the severity or duration of HF [3]. In summary, the amount of time to diagnosis and treatment plays a crucial role throughout the entire journey in HF patients, and we have an urgent need to better identify HF patients and provide adequate treatment in time. Current HF guidelines have also highlighted the importance of early diagnosis and treatment for HF patients to reduce disease progression and improve prognosis [4].

Many risk factors have been recognized for HF, most of which are associated with advancing age and MM level. Systemic aging has been acknowledged to influence various physiological processes contributing to structural and functional decline in cardiac tissue [5, 6]. Metabolic aging propels intricate shifts in lipid and glucose metabolism, leading to lipid accumulation, insulin resistance, and mitochondrial dysfunction within cardiomyocytes, contributing to an increased incidence of left atrial dilation, atrial fibrillation, myocardial fibrosis, left ventricular hypertrophy, and elevated susceptibility to chronic HF in the elderly population [7, 8]. Other age-related comorbidities like coronary artery disease (CAD), atherosclerosis, obesity, hypertension, diabetes, and chronic kidney disease (CKD) could further exacerbate HF [9]. CAD and atherosclerosis cause myocardial ischemia and dysfunction, while CKD-related fluid overload and uremic toxins aggravate HF through systemic inflammation and neurohormonal renin-angiotensin-aldosterone system (RAAS) activation [10, 11]. Hypertension drives cardiac hypertrophy and fibrosis, resulting in both systolic and diastolic dysfunction [12]. Obesity-associated insulin resistance, hyperlipidemia, and inflammation contribute to cardiac dysfunction through metabolic disturbances [13]. The pathogenesis of the increased HF in elderly individuals could also partly be explained by long-term exposure to genomic, oxidative, epigenetic, autophagic, inflammatory, and regenerative stresses [14].

Since HF is strongly associated with MM level, we believe that the incidence of HF and its common comorbidities have a mutual effect on each other [15]. For example, the patients with the combination of HF and type 2 diabetes (DM) were reported to have 37% (95% CI 35.9–38.1) incidence of a third condition within five years. Meanwhile, HF patients with CKD as a comorbidity only had 8.7% (95% CI 8.4–9.0) incidence of a third condition within five years, most likely due to their high mortality rate (51.6%) [16]. Both CKD and DM are associated with an increased incidence of HF [17].

In this study, we aimed to identify individuals at high risk of developing HF over a two-year period using widely available information as risk factors for HF.

## Methods

### Data source and measurements

The Region Skåne healthcare register in southern Sweden provided data that contains anonymized registry information from the study population, including gender, age, diagnostic data, and SES. Data were collected on diagnoses at each consultation across all primary healthcare centers (PHCs) and secondary healthcare providers.

HF was diagnosed following the diagnosis criteria according to ESC (European Society of Cardiology) guidelines. The three criteria required for HF diagnosis were the following: typical clinical symptoms, such as fatigue, dyspnea, exertional intolerance, and edema of the lower body; objective findings of impaired cardiac function on echocardiography, myocardial scintigraphy, magnetic resonance tomography, or other imaging; and an elevated BNP value [18]. Diagnoses were recorded according to the International Statistical Classification of Diseases and Related Health Problems version 10 (ICD 10) for a period of 18 months prior to the last week of 2015. HF was identified if the diagnosis code I50 was recorded, including the subtypes I500 - I501 and I509, which comprised right-sided HF and different EF categories of left-sided HF.

### Multimorbidity

A method developed by A Calderòn-Larrañaga et al. at the Aging Research Centre in Stockholm was used to identify chronic conditions [19]. They analyzed the full list of ICD-10 codes on a four-digit level to define whether a diagnosis is chronic or not in an elderly population. To determine if a condition was chronic or not, the following key features were identified and discussed concerning their pertinence and sustainability in older populations: duration, course, reversibility, treatment, and consequences. All information about diagnoses was obtained from the electronic medical record database in the county council in Region Skåne. MM level was then estimated by counting the number of chronic conditions per patient and dividing them into 10 groups. Patients with two chronic conditions or more were considered multimorbid, i.e. MM level 4 had four chronic conditions and MM level 10 had at least 10 chronic conditions.

### Socioeconomic status

SES was measured as Care Need Index (CNI) [20] to divide the PHCs into 10 groups depending on their CNI. CNI is based on different measures of a group, in this case the patients listed at the PHCs in southern Sweden (Region Skåne). Most people are listed at the PHCs in their neighborhood, which has an average CNI level. CNI 1 was assigned to patients listed at PHCs who belonged to the most socioeconomically affluent percentile, and CNI 10 to those who belonged to the most socioeconomically deprived percentile [20].

### Statistical analyses

We analyzed the population living in Region Skåne, of whom only those aged 20 years and older without a HF diagnosis were included. From a total of 961,190 study participants living in Region Skåne at the end of 2015 (31st December 2015), 50.9% were women and 49.1% were men. The variables gender, age, MM level, and SES characterized the study population.

We used frequencies, percentages, and cross-tabulations for descriptive analysis. Logistic regression was used to estimate the OR of a HF diagnosis within two years using multivariate models. The primary outcome was to identify individuals at high risk of HF diagnosis within two years (until 31st December 2017). A p-value of < 0.05 was considered statistically significant. Linear predictions were made based on models by adding variables in steps, e.g. gender, age, MM level, and SES (Model 1 - Model 4). Each model was compared with the previous model using a likelihood-ratio test, which were all statistically significant.

ROC and AUC were used to compare the different models’ predictive value of the four variables for probability of HF diagnosis within two years [21]. A heatmap illustrated the predicted percentage probabilities according to age and MM level. To find the optimal cut-off point to measure sensitivity and specificity, the Youden method was applied. Positive and negative predictive values were calculated using the prevalence in the whole study population and for those aged 70 and older.

We used STATA version 19.5 (Stata Corporation, Texas, USA) for statistical analyses.

## Results

The total prevalence of HF in the study population was approximately 2.0%. The prevalence of diagnosed HF within two years according to gender, age, MM level, and CNI level is presented in Table 1. 47.9% of the HF patients were women, and 52.1% were men. The prevalence of HF increased continuously with advancing age, from 0.2% in the age group 20 to 42.3% in the age group 80 (Table 1). MM level 4 had the highest prevalence of HF patients (13.0%), which tended to diminish with increasing MM level, although the study population decreased steadily with advancing MM level. CNI 6 had the highest prevalence of HF patients (11.6%), and CNI 10 had the lowest prevalence of HF (7.0%) (Table 1). 57.7% of the diagnosed HF patients within two years had hypertension, and 25.5% had ischemic heart disease (Appendix).

**Table 1:** Prevalence of diagnosed heart failure in Region Skåne within two years, categorized by different variables

Variables	No HF	HF	Total	*P*-value
N	949,265 (98.8%)	11,925 (1.2%)	961,190 (100%)	
gender				
women	483,691 (51.0%)	5707 (47.9%)	489,398 (50.9%)	< 0.001
men	465,574 (49.0%)	6218 (52.1%)	471,792 (49.1%)	
age				
20	180,788 (19.0%)	32 (0.3%)	180,820 (18.8%)	< 0.001
30	169,901 (17.9%)	73 (0.6%)	169,974 (17.7%)	
40	174,453 (18.4%)	260 (2.2%)	174,713 (18.2%)	
50	150,641 (15.9%)	743 (6.2%)	151,384 (15.7%)	
60	144,297 (15.2%)	2059 (17.3%)	146,356 (15.2%)	
70	88,949 (9.4%)	3712 (31.1%)	92,661 (9.6%)	
80	40,236 (4.2%)	5046 (42.3%)	45,282 (4.7%)	
MM level				
0	420,620 (44.3%)	960 (8.1%)	421,580 (43.9%)	< 0.001
1	181,730 (19.1%)	885 (7.4%)	182,615 (19.0%)	
2	119,003 (12.5%)	1143 (9.6%)	120,146 (12.5%)	
3	79,575 (8.4%)	1409 (11.8%)	80,984 (8.4%)	
4	54,212 (5.7%)	1554 (13.0%)	55,766 (5.8%)	
5	36,172 (3.8%)	1501 (12.6%)	37,673 (3.9%)	
6	23,488 (2.5%)	1251 (10.5%)	24,739 (2.6%)	
7	14,728 (1.6%)	1063 (8.9%)	15,791 (1.6%)	
8	8835 (0.9%)	803 (6.7%)	9638 (1.0%)	
9	5047 (0.5%)	471 (3.9%)	5518 (0.6%)	
10	5855 (0.6%)	885 (7.4%)	6740 (0.7%)	
CNI level				
1	108,279 (11.4%)	1275 (10.7%)	109,554 (11.4%)	< 0.001
2	107,206 (11.3%)	1349 (11.3%)	108,555 (11.3%)	
3	91,153 (9.6%)	1208 (10.1%)	92,361 (9.6%)	
4	84,007 (8.8%)	1174 (9.8%)	85,181 (8.9%)	
5	72,619 (7.7%)	954 (8.0%)	73,573 (7.7%)	
6	99,877 (10.5%)	1385 (11.6%)	101,262 (10.5%)	
7	84,201 (8.9%)	1157 (9.7%)	85,358 (8.9%)	
8	110,628 (11.7%)	1304 (10.9%)	111,932 (11.6%)	
9	110,158 (10.6%)	1285 (10.8%)	101,443 (10.6%)	
10	91,137 (9.6%)	834 (7.0%)	91,971 (9.6%)	

The variable with the highest OR for HF within two years was age, and the second highest OR was MM level (Table 2). 84.4% of those who developed HF had MM two years earlier. The OR of a HF diagnosis within two years rose sharply with advancing age and MM level. The OR in age group 20 was 1 and increased to 332.60 in age group 80 (95% CI 227.22–458.01). The OR in MM level 0 was 1 and reached 5.16 in MM level 10 (95% CI 4.58–5.81). Men had a higher OR for HF than women (OR 1.55, 95% 1.49–1.62, vs. OR 1). The most socioeconomically affluent CNI percentile had the lowest OR of HF (OR 1). In contrast, the most socioeconomically deprived group had the highest OR (OR 1.40, 95% CI 1.28–1.54), when adjusted for gender, age, and MM level. All values were statistically significant, with the exception of CNI 2.

**Table 2:** Odds ratios of heart failure diagnosis within two years, depending on different variables

Variables	OR (HF)	95% CI	*P* - value
gender			
women	1		
men	1.55	1.49–1.61	< 0.01
age			
20	1		
30	2.32	1.53–3.51	< 0.01
40	7.51	5.20–10.84	< 0.01
50	21.11	14.80–30.09	< 0.01
60	50.13	35.30–71.20	< 0.01
70	117.38	82.68–166.64	< 0.01
80	332.60	227.22–458.01	< 0.01
MM level			
0	1		
1	1.26	1.15–1.38	< 0.01
2	1.65	1.51–1.80	< 0.01
3	2.11	1.94–2.30	< 0.01
4	2.64	2.43–2.88	< 0.01
5	3.21	2.94–3.49	< 0.01
6	3.58	3.28–3.92	< 0.01
7	4.41	4.02–4.85	< 0.01
8	5.21	4.71–5.77	< 0.01
9	5.16	4.58–5.81	< 0.01
10	7.98	7.21–8.83	< 0.01
CNI level			
1	1		
2	1.07	0.99–1.16	> 0.05
3	1.16	1.07–1.26	< 0.01
4	1.20	1.10–1.30	< 0.01
5	1.23	1.13–1.34	< 0.01
6	1.15	1.07–1.25	< 0.01
7	1.24	1.14–1.34	< 0.01
8	1.18	1.09–1.28	< 0.01
9	1.36	1.25–1.47	< 0.01
10	1.40	1.28–1.54	< 0.01

HF = heart failure, CI = confidence interval, MM = multimorbidity, CNI = Care Need Index, OR = odds ratio

**Fig. 1:**
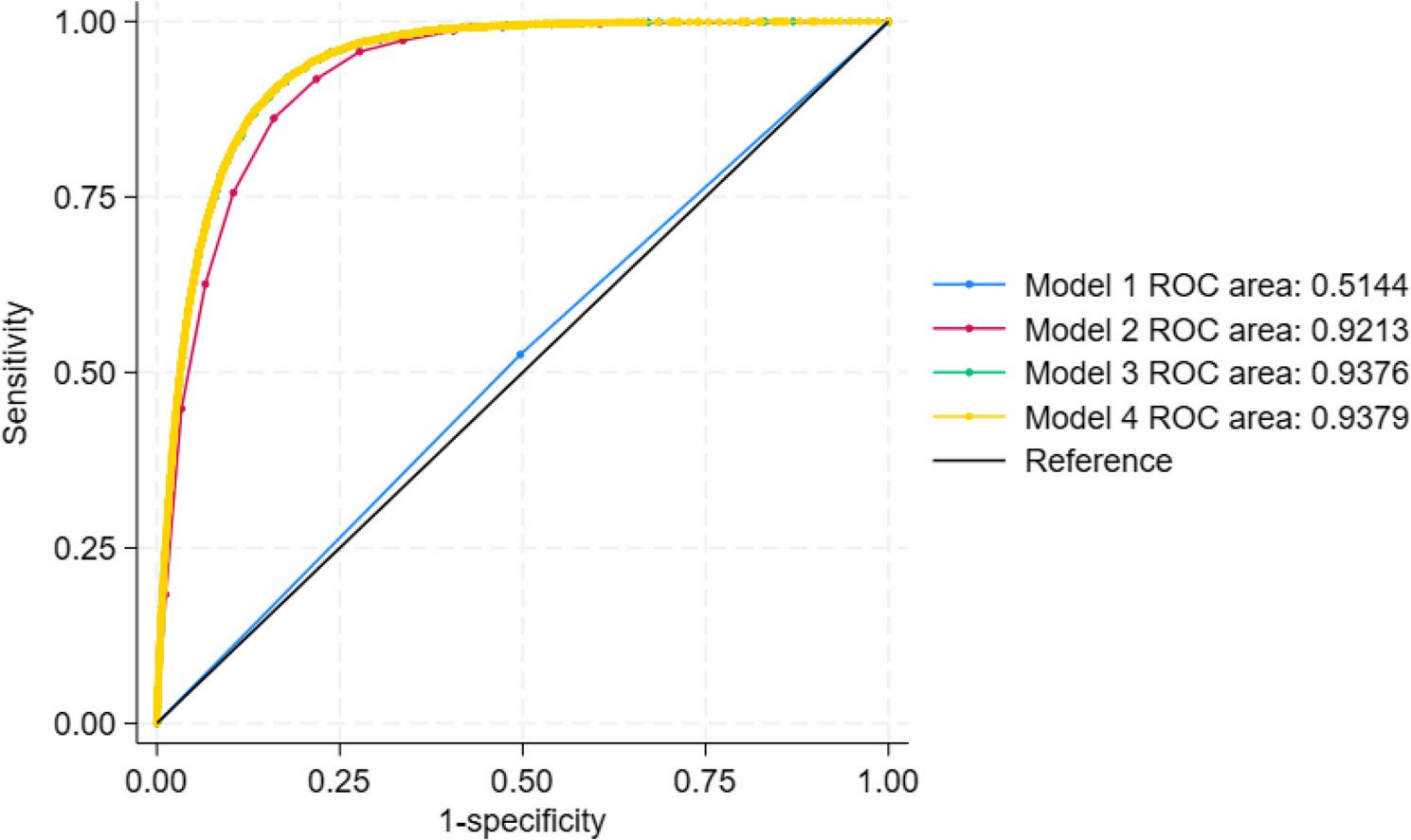
ROC (Receiver Operating Characteristic) curves comparing four models of different complexity, Models 1–4. Model 1 = gender. Model 2 = gender and age. Model 3 = gender, age, and multimorbidity level. Model 4 = gender, age, multimorbidity level, and socioeconomic status (CNI level)

ROC included the variables gender, age, MM level, and SES in steps, Model 1–4. Each model was compared with the previous model using a log likelihood test, which generated an increasing AUC from 0.51 to 0.94 (Fig. 1). AUC for gender (Model 1) was 0.5144; for gender and age (Model 2), it increased to 0.9213; and for gender, age, and MM level (Model 3), it increased further to 0.9376. For the combination of all four variables, gender, age, MM level, and SES (Model 4), the AUC reached 0.9379 (Fig. 1).

**Fig. 2:**
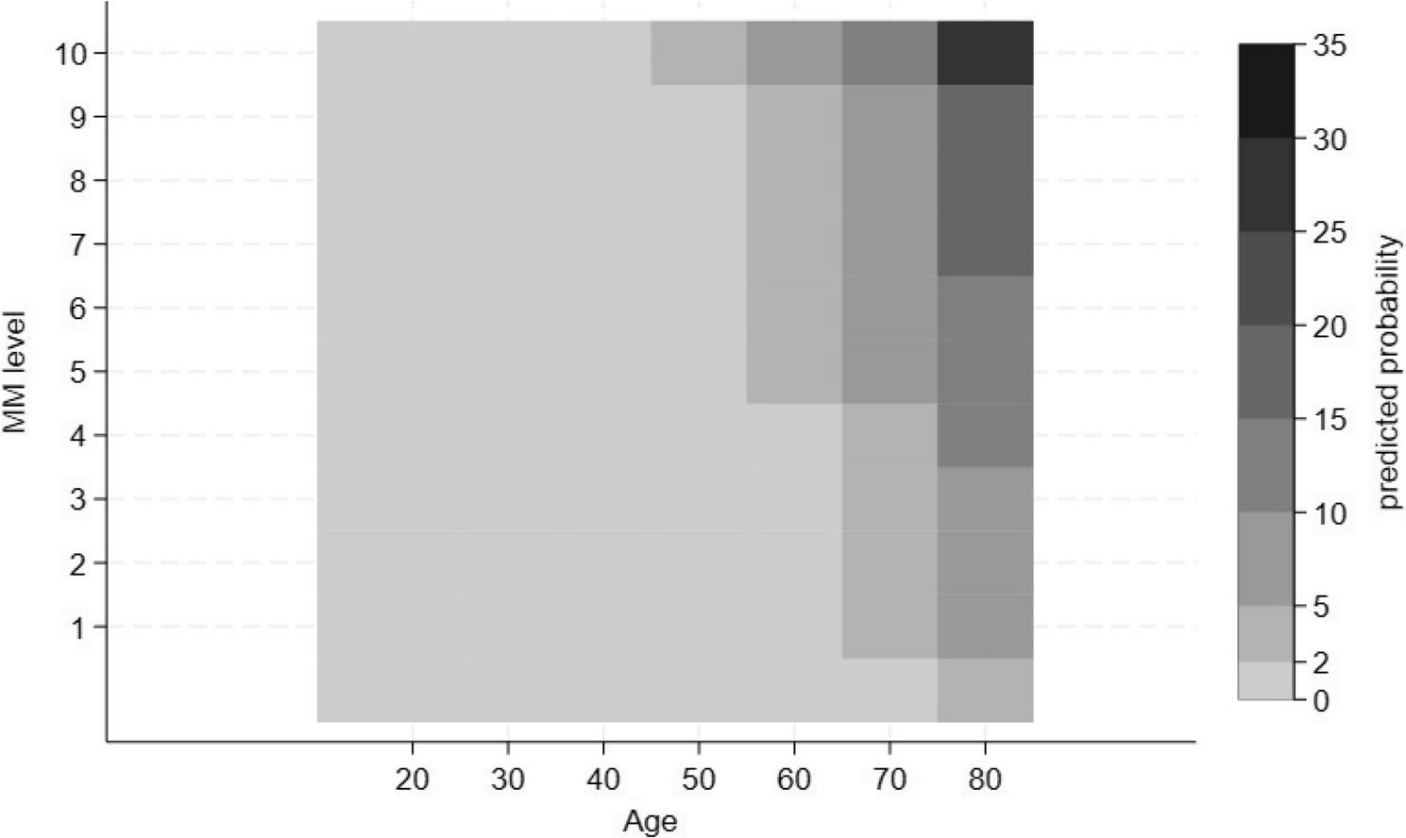
Heatmap of the predicted percent probabilities (Model 4) for heart failure diagnosis according to multimorbidity levels

The heatmap in Fig. 2 presents the predicted percentage probabilities when all four variables were included (Model 4), which were approximately highest at the combination of age over 70 and MM level five or higher.

When all four variables were in the model, an optimal cutoff point according to Youden was established at 1.15% predicted probability with a sensitivity of 87.69% and specificity of 78.48%. The positive predictive value was 4.78%, and the negative predictive value was 99.81% for the whole adult population; for those aged 70 years and older, it was 21.02% and 98.99%; and for those aged 80 years and older, it was 33.62% and 98.09%, respectively.

## Discussion

The total prevalence of HF in the study population was approximately 2.0%, which was comparable with the prevalence in Sweden and other Western countries [15, 22, 23]. Age was found to be the major factor to predict the probability of a HF diagnosis within two years in our study, followed by MM level. Gender and SES added marginally to the predictive value. The difference in the predictive value between these variables correlated with their OR of HF within two years. ROC generated an increasing AUC by adding the four variables in steps and reached an AUC of 0.9379 when all four variables were included. The positive and predictive values were 4.78%, and the negative predictive value was 99.81% for the whole adult population; for those aged 70 years and older, it was 21.02% and 98.99%; and for those aged 80 years and older, it was 33.62% and 98.09%, respectively.

Interestingly, the difference in OR of HF diagnosis was obvious between the genders when adjusted for age, MM, and SES, which is in line with our published results from this study population: the elderly socioeconomically deprived men with a high MM level were more prone to develop HF [24]. Ischemic heart disease, which is recognized as the most common etiology of HF in Western countries, affects men to a greater extent and consequently contributes to the higher OR of HF diagnosis in men than women [25, 26].

Our previous cross-sectional study on this study population revealed that the probability of HF increased substantially with advancing age and MM level, which certainly explains the highest OR of HF diagnosis within two years in the age group 80 [15]. MM level 4 had the highest prevalence of HF patients (13.0%), which tended to diminish with increasing MM level. This finding is congruent with our prior report of this study population, which stated that only 21.70% of all HF patients belonged to the patient group with 10 chronic conditions or more, meanwhile 58.18% of all HF patients belonged to the patient group with 4–9 chronic conditions [15]. These results lead us to suspect that HF patients with high MM burden have a poor prognosis. The most socioeconomically deprived group in our study population had the highest probability for HF, and only 33% were over 50 years old, thus indicating that these individuals have more potential to improve their outcomes than the more socioeconomically affluent population, when hypothesizing that advancing age is a strong risk factor for HF [15].

The very pronounced increase in OR of HF diagnosis with advancing age is most likely a synergetic effect of MM level, socioeconomic deprivation, and the age-related chronic HF process like inflammation, mitochondrial dysfunction, senescence, and declining cardiomyocyte regeneration [27]. A cohort study including 24,675 participants without HF diagnosis had stratified the study population by age into young (< 55 years; *n* = 11,599), middle-aged (55–64 years; *n* = 5,587), old (65–74 years; *n* = 5,190 and elderly (≥ 75 years; *n* = 2,299) individuals [28]. Over the median follow-up time of 12.7 years, 1% of young, 5% of middle-aged, 10% of old, and 18% of elderly participants developed HF. 32% of the HF cases in young participants were classified as HFpEF compared with 43% in elderly participants [28]. Risk factors, including diabetes, hypertension, current smoking history, and previous myocardial infarction, implicated a greater relative risk in younger compared with older participants. For example, hypertension caused more than double the risk of HF development in young participants (HR 3.02, 95% CI 2.10–4.34) than in elderly participants (HR 1.43, 95% CI 1.13–1.81) [28]. These younger participants had a lower MM burden compared to the elderly, which could explain their lower incidence of HF and a higher relative risk of HF development from each comorbidity. The absolute risk of developing HF was lower in younger than in older participants with and without risk factors, which could be explained by the age-related changes in the myocardium in elderly individuals, fostering HF development [28].

A wide variety of comorbidities are expected to contribute differently to HF diagnosis, which have more impact than SES and gender, but less than advancing age. Recognizing MM as an important modifiable risk factor for HF could guide us to target the individuals at high risk for HF in order to initiate both lifestyle changes and medical therapy to prevent HF.

### Strengths and limitations

The main strength is that the similar results compared with the calculations based on the same study population from our previous studies [15, 24]. AUC was high (0.94), although only four easily available variables were included in the ROC analysis. These results could guide us to differentiate and prioritize many risk factors on the way to identifying individuals at high risk for HF. The study population constitutes a heterogenous ethnicity, which is representative of many Western countries.

This study has several limitations. We did not have access to the ejection fraction (EF) data and thus not to the different subtypes of HF, i.e., with reduced EF (HFrEF), moderately reduced EF (HFmrEF), or preserved EF (HFpEF), which have different associations with gender, age, MM level, and SES. Some patients with typical HF symptoms have not been investigated with the conventional methods upon contact with healthcare, which causes a delayed registration of this diagnosis. The combination of different comorbidities could be more clearly defined in order to facilitate the identification of typical HF patients. For example, CAD, hypertension, chronic obstructive pulmonary disease (COPD), atrial fibrillation, CKD, and obesity likely have more impact on HF development than other comorbidities like cataract, arthritis, and dementia. Our results could be more accurate if only the strong risk factors were included in the MM level. Some risk factors for HF like obesity or hypertension, which do not cause immediate disability, could easily be neglected by the patients, resulting in a delayed diagnosis and lower MM level. The severity of the comorbidities, likewise the quality of their treatments, could also have a different impact on HF development. Our calculations did not consider the continuous increase of the population living in Region Skåne. Another source of error could be the dynamic nature of CNI classification during the study period, because all patients can be listed at PHCs of their own choice and not always at the place of their neighborhood.

Each model was compared with the previous model using a likelihood ratio test, but the model performance was evaluated only within the dataset without any validation procedure. Thus, the reported AUC should be interpreted cautiously because other confounders, like smoking history or heredity, could also have an influence on HF development.

## Conclusion

Age was the major factor to predict HF diagnosis within two years in our study, followed by MM, gender, and SES. These findings may help identify population groups at increased risk of HF in whom targeted case-finding strategies could be evaluated in future studies.

## Data Availability

Region Skåne County council provided anonymized data of the study population. The datasets used and/or analysed during the current study are available from the corresponding author on reasonable request.

## Appendix

**Table 3:** Prevalence of hypertension in diagnosed heart failure patients within two years

variables	no hypertension (prevalence)	hypertension (prevalence)	total (prevalence)	*P*-value
N	5046 (42.3%)	6879 (57.7%)	11,925 (100%)	
gender				
women	2261 (44.8%)	3446 (50.1%)	5707 (47.9%)	< 0.001
men	2785 (55.2%)	3433 (49.9%)	6218 (52.1%)	
age				
20	31 (0.6%)	1 (0.0%)	32 (0.3%)	< 0.001
30	64 (1.3%)	9 (0.1%)	73 (0.6%)	
40	188 (3.7%)	72 (1.0%)	260 (2.2%)	
50	441 (8.7%)	302 (4.4%)	743 (6.2%)	
60	947 (18.8%)	1112 (16.2%)	2059 (17.3%)	
70	1385 (27.4%)	2327 (33.8%)	3712 (31.1%)	
80	1990 (39.4%)	3056 (44.4%)	5046 (42.3%)	
MM level				
0	960 (19.0%)	0 (0.0%)	960 (8.1%)	< 0.001
1	718 (14.2%)	167 (2.4%)	885 (7.4%)	
2	727 (14.4%)	416 (6.0%)	1143 (9.6%)	
3	664 (13.2%)	745 (10.8%)	1409 (11.8%)	
4	560 (11.1%)	994 (14.4%)	1554 (13.0%)	
5	472 (9.4%)	1029 (15.0%)	1501 (12.6%)	
6	341 (6.8%)	910 (13.2%)	1251 (10.5%)	
7	221 (4.4%)	842 (12.2%)	1063 (8.9%)	
8	167 (3.3%)	636 (9.2%)	803 (6.7%)	
9	89 (1.8%)	382 (5.6%)	471 (3.9%)	
10	127 (2.5%)	758 (11.0%)	885 (7.4%)	
CNI level				
1	588 (11.7%)	687 (10.0%)	1275 (10.7%)	< 0.001
2	538 (10.7%)	811 (11.8%)	1349 (11.3%)	
3	523 (10.4%)	685 (10.0%)	1208 (10.1%)	
4	498 (9.9%)	676 (9.8%)	1174 (9.8%)	
5	350 (6.9%)	604 (8.8%)	954 (8.0%)	
6	598 (11.9%)	787 (11.4%)	1385 (11.6%)	
7	468 (9.3%)	689 (10.0%)	1157 (9.7%)	
8	560 (11.1%)	744 (10.8%)	1304 (10.9%)	
9	550 (10.9%)	735 (10.7%)	1285 (10.8%)	
10	377 (7.4%)	461 (6.7%)	834 (7.0%)	

## Appendix

**Table 4:** Prevalence of ischemic heart disease (IHD) in diagnosed heart failure patients within two years

variables	IHD (prevalence)	IHD (prevalence)	total (prevalence)	*P*-value
N	8884 (74.5%)	3041 (25.5%)	11,925 (100%)	
gender				
women	4533 (51.0%)	1174 (38.6%)	5707 (47.9%)	< 0.001
men	4351 (49.0%)	1867 (61.4%)	6218 (52.1%)	
age				
20	30 (0.3%)	2 (0.1%)	32 (0.3%)	< 0.001
30	68 (0.8%)	5 (0.2%)	73 (0.6%)	
40	232 (2.6%)	28 (0.9%)	260 (2.2%)	
50	608 (6.8%)	135 (4.4%)	743 (6.2%)	
60	1582(17.8%)	477 (15.7%)	2059 (17.3%)	
70	2707 (30.5%)	1005 (33.0%)	3712 (31.1%)	
80	3657 (41.2%)	1389 (45.7%)	5046 (42.3%)	
MM level				
0	960 (10.8%)	0 (0.0%)	960 (8.1%)	< 0.001
1	846 (9.5%)	39 (1.3%)	885 (7.4%)	
2	1029 (11.6%)	114 (3.7%)	1143 (9.6%)	
3	1162 (13.1%)	247 (8.1%)	1409 (11.8%)	
4	1164 (13.1%)	390 (12.8%)	1554 (13.0%)	
5	1059 (11.9%)	442 (14.5%)	1501 (12.6%)	
6	862 (9.7%)	389 (12.8%)	1251 (10.5%)	
7	673 (7.6%)	390 (12.8%)	1063 (8.9%)	
8	470 (5.3%)	333 (11.0%)	803 (6.7%)	
9	247 (2.8%)	224 (7.4%)	471 (3.9%)	
10	412 (4.6%)	473 (15.6%)	885 (7.4%)	
CNI level				
1	971 (10.9%)	304 (10.0%)	1275 (10.7%)	0.153
2	989 (11.1%)	360 (11.8%)	1349 (11.3%)	
3	908 (10.2%)	300 (9.9%)	1208 (10.1%)	
4	854 (9.6%)	320 (10.5%)	1174 (9.8%)	
5	720 (8.1%)	234 (7.7%)	954 (8.0%)	
6	1004 (11.3%)	381 (12.5%)	1385 (11.6%)	
7	853 (9.6%)	304 (10.0%)	1157 (9.7%)	
8	974 (11.0%)	330 (10.9%)	1304 (10.9%)	
9	990 (11.1%)	295 (9.7%)	1285 (10.8%)	
10	621 (7.0%)	213 (7.0%)	834 (7.0%)	

## Abbreviations

AUC: Area under the curve
CAD: coronary artery disease
CKD: chronic kidney disease
CNI: Care Need Index
COPD: chronic obstructive pulmonary disease
DM: diabetes mellitus
EF: ejection fraction
HF: heart failure
HR: hazard ratio
MM: multimorbidity
OR: odds ratio
PHC: Primary healthcare center
ROC: Receiver Operating Characteristic
SES: socioeconomic status
